# Heartbeats Do Not Make Good Pseudo-Random Number Generators: An Analysis of the Randomness of Inter-Pulse Intervals

**DOI:** 10.3390/e20020094

**Published:** 2018-01-30

**Authors:** Lara Ortiz-Martin, Pablo Picazo-Sanchez, Pedro Peris-Lopez, Juan Tapiador

**Affiliations:** 1 Department of Computer Science, Carlos III University of Madrid, 28911 Leganés, Spain; 2 Department of Computer Science and Engineering, Chalmers University of Technology∣Gothenburg University, 41296 Gothenburg, Sweden

**Keywords:** randomness, authentication, privacy, implantable medical devices, inter-pulse intervals, biometric

## Abstract

The proliferation of wearable and implantable medical devices has given rise to an interest in developing security schemes suitable for these systems and the environment in which they operate. One area that has received much attention lately is the use of (human) biological signals as the basis for biometric authentication, identification and the generation of cryptographic keys. The heart signal (e.g., as recorded in an electrocardiogram) has been used by several researchers in the last few years. Specifically, the so-called Inter-Pulse Intervals (IPIs), which is the time between two consecutive heartbeats, have been repeatedly pointed out as a potentially good source of entropy and are at the core of various recent authentication protocols. In this work, we report the results of a large-scale statistical study to determine whether such an assumption is (or not) upheld. For this, we have analyzed 19 public datasets of heart signals from the Physionet repository, spanning electrocardiograms from 1353 subjects sampled at different frequencies and with lengths that vary between a few minutes and several hours. We believe this is the largest dataset on this topic analyzed in the literature. We have then applied a standard battery of randomness tests to the extracted IPIs. Under the algorithms described in this paper and after analyzing these 19 public ECG datasets, our results raise doubts about the use of IPI values as a good source of randomness for cryptographic purposes. This has repercussions both in the security of some of the protocols proposed up to now and also in the design of future IPI-based schemes.

## 1. Introduction

eHealth is a relatively novel term that is commonly used to refer to healthcare services delivered through (or making an extensive use of) technology and telecommunications systems. eHealth can be seen as a special subset of the *Internet of Things (IoT)*, where “things” are essentially sensors that are constantly gathering information about the medical condition of a subject. Additionally, when these sensors are placed in, on or around the human body to monitor anywhere and anytime the vital signs of the bearer, it is said to be part of a *Body Area Network (BAN)* (also known as a *Body Sensor Network (BSN)*). BAN devices can communicate with a central device (also known as a hub, which is commonly implemented by a smartphone) with Internet connectivity, and in the near future, all these devices will be able to interact directly between each other.

Information gathered by a BAN, which may contain highly sensitive data privacy-wise, is usually shared with other devices in the network and can also be sent to public servers in order to be accessible by different people such as medical staff, the user’s personal trainer or just for private purposes. It has been thought that *Implantable Medical Device (IMD)* such as pacemakers, insulin pumps or cochlear implants were the only devices in charge of measuring biological information. However, there are many other gadgets such as smartphones, wristbands or even smartwatches that can be used to sense some vital signs of the bearer without interfering in her/his life.

The security of this network and the gathered sensitive data have been identified as comprising one of the most challenging tasks by the research community [1,2,3] before deploying it in a real scenario. As an example, imagine someone who is equipped with sensors whose information is shared via wireless; it could be easy for an attacker to sniff the communication channel in order to listen to the transmitted packages and get some knowledge about the bearer. Therefore, new cryptographic protocols are needed not only to protect the user’s identity, but also to protect the integrity of the patient’s medical data [4,5].

Biometrics refers to the identification and authentication methods that, using biological signals, can identify or validate the identity of a person. In the few last years, several works have been focused on biometric authentication and identification [6,7,8]. This kind of authentication system has great potential because each biological trait must be universal, collectable, unobtrusive, permanent, unique and difficult to circumvent [9]. Biometric signals can be classified into physiological and behavioral signals [10]. Examples of physiological signals include face recognition, fingerprint, iris, *Electrocardiogram (ECG)*, *Electromyogram (EMG)* or *Galvanic Skin Response (GSR)*. Behavioral traits have also been proposed, such as the voice, signature, keystroke dynamics or lip movements, among others.

Biometrics have also been used to generate personal cryptographic keys [11] by using biological signals as a *Pseudorandom Number Generators (PRNG)*. Therefore, in order to check if a given sequence of numbers can be considered random, there are some well-known tests like Shannon’s entropy, the Monte Carlo test or the frequency test, among others. However, instead of using a subset of tests, there are some public suites like the ENT (ENT can be downloaded at http://www.fourmilab.ch/random/) test, a software published by the *National Institute of Standards and Technology Statistical Test Suite (NIST STS)*, the DieHard (Diehard can be downloaded at http://stat.fsu.edu/pub/diehard/) tool or TestU01 (TestU01 can be downloaded at http://simul.iro.umontreal.ca/testu01/tu01.html), software that are more likely to be used when evaluating the randomness property [12]. It is important to remark that the ENT test was initially for general purposes, whereas the other suites are focused on guaranteeing some security properties.

### Overview of Our Results

**Figure 1:**
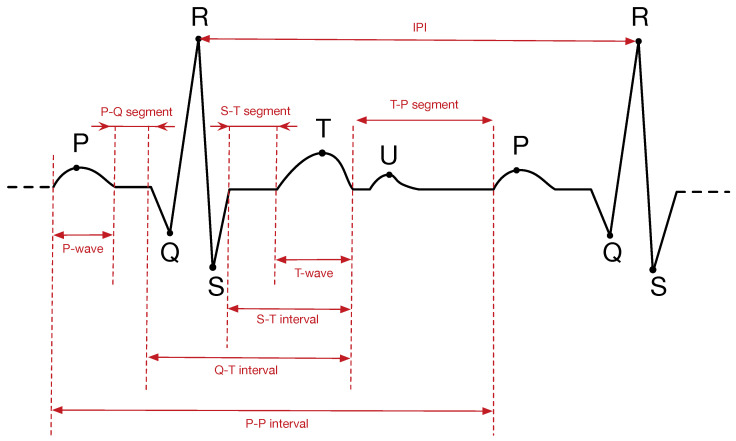
A typical electrocardiogram (ECG) signal and its main features: peaks (P, Q, R, S, T, U), waves, segments and intervals. IPI, Inter-Pulse Interval.

In the last few years, entropy analysis has been shown to be an effective mechanism to assist doctors in medical problems [13]. For instance, the analysis of brain images can help the detection of some brain diseases [14,15]. Another good example is the detection of cardiac problems through the analysis of ECG records [16,17,18]. In addition, and outside of the medical context, recent works have demonstrated that ECG signals can be also used as a source of entropy for security purposes [8,19,20,21]. In particular, this is done by calculating the *Inter-Pulse Interval (IPI)*, which is the time interval between two consecutive R-peaks of the ECG. If an arbitrary R-peak occurs at time $t_{R}\left(i\right)$, then IPI can be computed as the time difference between $t_{R(i)}$ and $t_{R(i-1)}$:$IPI_{\left(i\right)}=t_{R(i)}-t_{R(i-1)}$, as can be seen in Figure 1. We refer the reader to Section 2.2 for more details about the components of an ECG signal and to Section 3.2 for the IPI extraction algorithm.

Nowadays, apart from the common medical electrodes that record the ECG signal, there exists a myriad of devices equipped with dedicated sensors to measure the heart signal. For instance, measuring the heart rate can determine the efficiency of a workout or even the calories that someone has burned. In order to do so, the exercise machines used in gyms normally have some metallic areas located on the support bars that interpret small electrical signals passing through the skin. There are, however, some other wearable devices with *Photoplethysmographic (PPG)* sensors that record the blood pressure to obtain the heart beats, i.e., a device illuminates the skin with a light source like an LED to detect the changes in the light absorption. Nowadays, PPG monitors are usually found in most of the wristbands and smartwatches. Some other mechanisms like chest bands are commonly used by athletes when they are training or even in competitions to check their heart rates

Many authors have claimed that the *Least Significant Bits (LSBs)* of the IPI contain a high degree of entropy [6,8,19,22,23,24,25,26,27,28,29,30]. In addition, most of these authors use some public databases to prove this entropy property, and thus, with this method, the resulting bits can be considered as random numbers and can be part of key generation protocols in authentication procedures.

Recent IPI-based authentication, identification and key generation protocols (e.g., [25,28,29,30]) suffer from two main weaknesses. First, they only use measures of entropy to determine whether the generated cryptographic material (keys and other intermediate values, such as nonces) are random or not. Second, the datasets used in these works are rather small and, therefore, possibly not significant enough. Additionally, such datasets contain ECG signals obtained both from healthy subjects and others that suffer some heart-related pathology, and it is unclear whether this feature has some influence on the overall quality (i.e., randomness) of the derived bits. Some of these observations have been already raised in [12], in which the authors pointed out the need to perform a more sound assessment of the quality of the generated keys using larger datasets and additional randomness tests. Nevertheless, the code that the authors used to run these experiments is not available.

In this work, we overcome these weaknesses by performing an analysis of the randomness of 19 different public databases containing heart signals. Our contributions can be summarized as:

- We have downloaded 19 public databases with information about heart signals from different people. All datasets are taken from the Physionet repository (https://physionet.org/physiobank/database/#ecg) [31], which contains heart signals from both healthy volunteers and people with cardiac conditions. We then extracted the last four bits of the IPI of each person per database, thus creating a bit stream whose quality can be tested. In doing so, we attempt to address the gap detected in [12].
- We analyze all files independently to check if the ECG can be considered to be a good random number generator. To do so, two random number suites (ENT, general purpose, and NIST STS, security) have been run over all previously generated files. To the best of our knowledge, this is the first work that discusses how the ECG signal should be used in cryptographic protocols as a source of random numbers. Our scripts are made public (https://github.com/aylara/Random_ECG) to facilitate the replication of our results by other researchers.
- Contrary to prior proposals, we demonstrate that the ECG signal contains some degree of randomness, but its use in cryptographic applications is questionable. Some databases obtained reasonable results on either ENT or NIST STS. However, none of the tested databases obtained good results on both at the same time except the mitdb database.

The rest of the paper is organized as follows: Section 2 provides some background on biometric authentication according to ECG and the basic description of some random tests. Section 3 describes the evaluation of our implementations and a discussion of the results. This paper ends with some conclusions in Section 4.

## 2. Background

In this section, we provide some background on related work: biometric authentication, IPI-based authentication and key derivation protocols, as well as randomness tests.

### 2.1. Biometric Authentication

**Figure 2:**
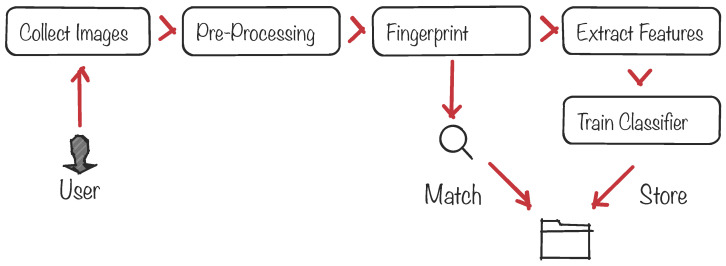
Architecture of a generic biometric recognition system.

Biometric protocols provide security services such as the authentication and identification of a given person among a large set of people. Figure 2 illustrates the standard pipeline of a biometric system, from the signal acquisition and preprocessing to the final decision-making process to identify/authenticate the subject. At the core of the system there is a pattern-matching process between a freshly-acquired template built from the subject’s signal and a previously-stored template. The matching process is usually done by defining an acceptance threshold and calculating the Hamming distance between both templates to decide whether the subject is or is not authenticated. The signal is usually acquired by sensors that can be located in, on or around the human body. Examples of well-known biometric signals include the iris [32], the fingerprint and face [33,34], the voice [35] and the ECG [36].

Biometric approaches have been combined with traditional cryptographic primitives in several ways, including the replacement of matching algorithms by secure versions [37,38], using biometric templates in *Secure Multiparty Computation (SMC)*, homomorphic encryption schemes [34,39,40] or with elliptic curves [41,42]. Apart from cryptographic proposals, the use of biometric signals to generate cryptographic keys has been widely studied in the literature (see, e.g., [6,22,23,24,25,26,27,28,29,30,43]). In most of these works, the authors obtain a biological signal from different sensors or devices, such as the Electroencephalogram (EEG), the PPG, the ECG or accelerometers, and check whether the signals can be considered random or not. To do so, the common practice is to extract some feature(s) from the signal and then run several randomness tests to validate the hypothesis.

Particularly, the use of IPIs has gained special attention in cryptographic applications as a random number generator; for instance, in [44,45,46] to generate a private key, in [6] to be part of an authentication protocol, in [47,48,49] as an alternative to classical key establishment protocols or in [50] as part of a proximity detection protocol. It is worth noting the transcendence that IPIs have in all the aforementioned scenarios and why the random number generation is crucial.

### 2.2. IPI-Based Security Protocols

Figure 1 shows a typical ECG trace. The signal contains six different peaks, known by the letters P, Q, R, S, T and U. Heartbeats are commonly measured as the time distance between two consecutive R-peaks. This is known as Inter-Pulse Interval (IPI), and several works published over the last decade have noted that the sequence of IPIs contains some entropy. To obtain such random bits, each IPI should be first quantized, i.e., represented in binary code using some coding scheme. Most works omit the details about the particular coding scheme used, which is quite unfortunate since this is a critical component for the entropy (or lack thereof) of the resulting binary sequence. One notable example is the work of Rostami et al. [6], which will be described in more detail in Section 3.2 since it is the coding scheme used in this paper.

The majority of the proposed works in this area, e.g., [25,27,28,29,30,51], conclude that the last four bits of each IPI can be used as a random number because of their high entropy. Thus, if an authentication protocol requires a 128-bit key to work, it would be necessary to acquire 32 IPIs (i.e., at least 33 consecutive R-peaks). Considering that a regular heart beats at 50–100 *bits per minute (bpm)*, the key generation process would take between 20 and 40 s. To prove that the extracted bits have a certain level of randomness, most works use either the common Shannon or Rényi entropies [29], which are not enough to claim the randomness property of a sequence of beats. Additionally, in [6,26,27,47,51,52], the authors remark about the same claims about the randomness of the IPIs by running the NIST STS battery of randomness tests, whereas in [8], the authors rely on the ENT suite. Table 1 summarizes the datasets that the existing works in this area have used. Additionally, in the last column, the number of executed tests can be seen where, for instance, NIST STS (5/15) means that authors have run five tests out of the 15 that the NIST STS suite has. Note that [52] is the only work where the authors ran all tests of which NIST STS is composed. We were not able to find the main reasons for running a subset of tests in the rest of the works that use NIST STS.

### 2.3. Randomness Tests

One key aspect of all IPI-based protocols is the assumption that some bits (four, typically) of each IPI are highly entropic. This condition is necessary, but not sufficient to guarantee the security of the protocol. In other words, high entropy does not necessarily imply randomness. Therefore, more sophisticated tests should be also applied to ensure that the values are indistinguishable from a random sequence.

In this paper, we have used the ENT [54] and NIST STS [12] suites to evaluate how good the generated random numbers are. In particular, ENT is a suite composed of the following tests: entropy, chi square, arithmetic mean, Monte Carlo and serial correlation coefficient statistical tests. Finally, ENT reports the overall randomness results after running the aforementioned tests. On the contrary, NIST STS is a suite made of fifteen statistical tests: frequency monobit and block tests, runs, longest run of ones in a block, binary matrix rank, the discrete Fourier transform (spectral) test, overlapping and non-overlapping template matching, Maurer’s universal statistical tests, linear complexity, serial, approximate entropy, cumulative sums, random excursions and random excursions variant tests. Finally, NIST STS reports a *p*-value that indicates whether the given sequence has passed each test or not.

For completeness, we refer the reader to the Appendix A where we provide a brief description of each one of the tests that form part of both the NEST and NIST STS suites.

## 3. The Randomness of IPI Sequences

This section describes our experiments to analyze the randomness of the IPI values and a discussion of the obtained results.

### 3.1. Dataset

For consistency with previous research in this area, we have first downloaded the mitdb, ptbdb and mghdb Physionet (the software package to access the data repository can be found at https://physionet.org/physiotools/wfdb.shtml) databases from [31], and we have tried to replicate the experimental setting used by both Rostami et al. in [6] and Xu et al. in [47]. The results, however, were impossible to reproduce due to the lack of information that the authors provide in the original papers. The downloaded databases contain the information of several subjects, and we do not know how the original experiments were run, e.g.,: (i) by acquiring the last 4 LSBs of the ECG of each one of the subjects and after that running (a subset) of the NIST STS tests per person; (ii) if the authors generated one single file with the information of all subjects belonging to the same database and then this file was used as the input of some of the NIST STS tests; or (iii) if the authors generated one single file with the information of all subjects of all databases and then they run (a subset of) the NIST STS tests.

Due to the fact that only one single value was given in [6] regarding the final results of the NIST STS and also that at a certain moment, the authors claim that they used an aggregate of different databases for the error generation, we assume that the authors used Approach (iii): they created one single file with the four LSBs of the IPI of different people belonging to different datasets. Nevertheless, we consider that this is not a realistic experiment because of the heterogeneous of the databases (see Table 2) as was also pointed out by [12]. On the contrary, the authors in [47] neither provide the achieved results of the NIST STS, nor do they say which database(s) they use for testing.

For these reasons, we have substantially extended this setting to 16 additional datasets of ECGs also present in the Physionet repository. All these datasets contain ECG records obtained from a variety of real subjects with different heart-related pathologies in many cases. Table 2 shows the main features of the 19 datasets used in this work. Furthermore, we have computed the median value of the extracted IPIs per file (person) per database. For instance, it is easy to argue that heart signals acquired from people equipped with holters (cdb) cannot be used to prove that the heart signal is random enough. Similar cases occur with the iafdb, ptdb or twadb databases with medians of 37, 68 and 87 IPIs, respectively.

In order to avoid the aforementioned problems and to allow other researchers to reproduce the results, we have split up the results into their corresponding databases. After that, we have extracted the four LSBs of each subject and ran the random tests (NIST STS and ENT suites) on each individual file (corresponding to each subject of each database) to evaluate how good the generated random numbers are. Finally, the results are grouped per pathology (database), and we give a percentage of the files (persons) that successfully passed the random tests.

### 3.2. IPI Extraction

Previous works in this area found out that the four LSBs of each IPI are highly entropic [6,47]. We replicated this process as follows. We first used a MATLAB script available at the Physionet repository (https://physionet.org/physiotools/software-index.shtml) to obtain the ECG signal for each record (person) in each one of the 19 datasets. We next applied the following steps:

1. Get the sampling frequency for each signal, which is available in an associated description record.
2. Run Pan–Tomkins’s QRS detection algorithm [74] over the ECG signal to extract the R-peaks.
3. Get the timestamp of each R-peak and calculate the difference between each pair of consecutive R-peaks to obtain the sequence of raw IPI values.
4. Apply a dynamic quantization algorithm to each IPI to decrease the measurement errors. This process consists of generating discrete values from an ECG (continuous signal).
5. Apply a Grey code to the resulting quantized IPI values to increase the error margin of the physiological parameters.
6. Extract the four LSB from each coded IPI value.

Each sequence of extracted bits per record of each dataset is stored in separate files for subsequent analysis.

**Figure 3:**
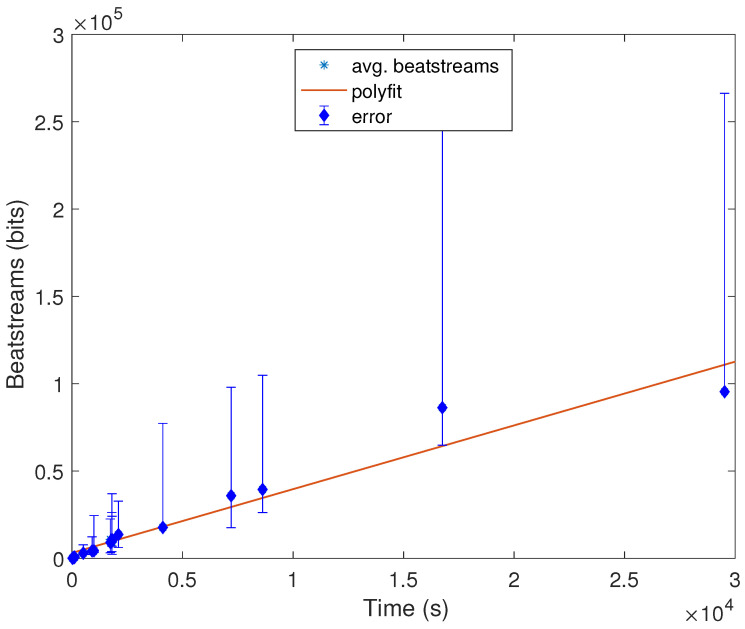
Statistical analysis of beat streams (in bits) and time (in seconds).

Additionally, we have also conducted one more experiment in MATLAB under a MacPro laptop with 4 Gb of RAM to estimate how long the signal should be to extract a stream of length *x* bits. To do so, we have computed the average number of IPIs and the length average of the signal of the nineteen databases. In Figure 3, these results can be seen from which we can conclude that the relation between time and the length of bits is linear, and for instance, after almost 4 h, we will have approximately 60,000 bits, which can be used as random numbers. It is also noticeable that these results are consistent with the hypothesis that in order to extract a valid cryptographic key, only a few seconds are enough. In other words, to generate a cryptographic key of 128 bits, a device should wait between 20 and 50 s to create that key. It is also remarkable that depending on the scenario, this time constraint might not be feasible to deploy; e.g., a person who is suffering from a heart attack cannot wait for a minute to authenticate her/his pacemaker with the caregiver device.

### 3.3. Measuring Randomness

In this section, we discuss the results of applying both the NIST STS and ENT test suites to the datasets discussed above.

#### 3.3.1. ENT

As described in Appendix A.1, the ENT suite is comprised of six tests of randomness. Table 3 shows the optimum value for each one of them. Along with this, we also provide two additional values for each test: (i) a threshold, which constitutes a more affordable value for each test since the optimal output is quite restrictive and most sequences would fail the tests otherwise; and (ii) the test result obtained for an input sequence consisting of a simple counter value from zero to $2^{14}$. The purpose of this experiment is just to demonstrate that the result of a single test cannot be used alone to claim evidence of randomness; see, e.g., the output achieved by the counting sequence for the entropy, the arithmetic mean, the serial correlation coefficient and the optimum compression.

The results obtained after applying the six ENT tests to each one of the files (persons) (with the IPIs of their ECG signals in our 19 datasets) can be seen in Table 4. Each cell in the table provides the percentage of persons who pass the test using the threshold shown in Table 3. For instance, in the case of the mitdb database, we have generated 46 files, belonging to 46 persons involved in this database, with a median of 1113 IPIs per file. The results for this database are that all persons pass both the entropy and optimum compression tests (100%), but none of them pass the chi square test (0%); 45 out of 46 pass the arithmetic mean and the serial correlation tests (97.83%); and 22 out of 46 pass the Monte Carlo value for π (47.83%).

Overall, the first noticeable observation is that these results are quite good across all datasets in the entropy, optimum compression and serial correlation, whereas for the chi square, the results are catastrophic. The situation is similar for Monte Carlo for the π test where all databases fail, but szdb, slpdb, edb and shareedb, achieving 71.43%, 74.47%, 60% and 55.52%, respectively. On the contrary, the arithmetic mean test achieves good results, but the vfdb and the cudb fail that test with 17% and 44.44%, respectively.

Looking at the results from a dataset perspective, we were not able to identify if there exists some correlation among the tests results with the information available to us (number of samples, sampling frequency, signal length, IPIs per file or characteristics of the subjects). See also the discussion provided later on in Section 3.4 for an additional analysis on this.

#### 3.3.2. NIST STS

In Appendix A.2, a description of all fifteen tests that comprise this suite can be read. As a common feature, all NIST STS tests are parameterized by a variable *n*, which means the length of bits of the processed bitstream. Additionally, some of the tests can also detect local non-randomness: the frequency test within block, overlapping and non-overlapping template matching, Maurer’s “universal statistical” test, linear complexity, serial and approximate entropy tests. These tests are also parameterized by a second variable denoted as *m* or *M*. Those tests that use the *m* parameter are mainly focused on detection of *m*-bit patterns in the stream, whereas those tests that use the *M* parameter check the distribution of the specific feature across *n*/*M* blocks of equal size (*M* bits). In Table 5, the minimum requirements in terms of length can be seen.

Furthermore, if we take into account the values of Table 2 regarding the length (median) of our datasets, we cannot run the original NIST STS with enough of a confidence level. The Physionet datasets are irregular in their size, with several of them having too small a size to be used with the original tests. In order to circumvent the length constraints that the original NIST STS has, we have used a variant [75] of the original software package.

Table 6 provides the success rate obtained for the 15 NIST STS tests for the files (subjects) of each dataset. In this case, we used the pass criteria included in each test, which are based on an analysis of the yielded *p*-values. In other words, *p*-values of less than 0.01 are considered rejected. Overall, the results are similar to those obtained for ENT, although in this case, the success rate is generally higher in most cases. Furthermore, there are substantial differences across datasets. For instance, iafdb, ptbdb and twadb obtain success rates higher than 80% in 12, 12 and 11 out of the 15 tests, respectively. In contrast, the performance of many datasets is considerably poor, with less than 50% of their records not passing a majority of the tests: see, for example, the cases of apnea-ecg and cudb (more than 50% of the records fail nine out of 15 tests); svdb (more than 50% of the records fail 19 out of 15 tests); edb, slpdb, szdb and vfdb (more than 50% of the records fail 11 out of 15 tests); mghdb (more than 50% of the records fail 12 out of 15 tests); and lspdb (more than 50% of the records fail 13 out of 15 tests). In the case of slpdb and szdb, the results are very deficient, with all signals in both datasets failing nine out of the 15 tests (i.e., 0% of success rate).

In terms of performance against individual tests, the results are rather diverse, with a few exceptions. The case of the linear complexity test stands out, as most datasets exhibit an extremely poor result. This suggests the existence of patterns that can be modeled by linear prediction functions, which undoubtedly implies predictability. Similarly, most datasets perform badly in the monobit and block frequency tests, which reveals a non-negligible imbalance of zeroes and ones (monobit frequency) and, more generally, all possible *M*-block bit patterns (block frequency).

Finally, it is worth noting that there seems to be some correlation among the test results, particularly for datasets that do and do not perform well. Consider, for example, the case of cdb (11/15), iafdb (13/15), ptdb (13/15), twadb (13/15) and cebsdb (14/15), which obtain extremely good results (at least passing 11 out of 15 tests in the worst case, i.e., cdb) for all tests. All these databases have in common that the median number of IPIs is less than 175; note that an IPI is made of four bits. Contrarily, shareedb (2/15), apnea-ecg (3/15), mghdb (3/15), edb (4/15), slpdb (4/15), szdb (4/15) and vfdb (4/15) achieve very poor results (only passing four out of 15 in the best case) in the NIST STS, having median of 46,910, 15,786, 2426, 4405, 11,517, 4439 and 1800 IPIs, respectively.

### 3.4. Discussion

In Table 7, a summary of all tested databases can be seen with the typology of each dataset in order to find out some relations between them. Notice that if we analyze the results on average, all databases achieve reasonable results in the ENT suite, whereas eight out of 19 pass the NIST STS tests. Nevertheless, this is not true at all, as we can see in Table 4 that none of the tests pass the chi square test, which is crucial due to this test checking if the sequence is random or not [54]. Moreover, the Monte Carlo test achieves 36.45%, i.e., only the edb, shareedb, slpdb and szdb databases pass the Monte Carlo test.

It has been previously pointed out (see Table 2) that many authors only use the mitdb dataset, for which it is true that it passes most of the tests of both suites, but not all of them. Thus, it is not a real assumption to claim that ECG can be considered to be random only by taking the entropy results. We have proven that a counter achieves similar results, and it is well known that it cannot be used as a random generator (Table 3).

**Figure 4:**
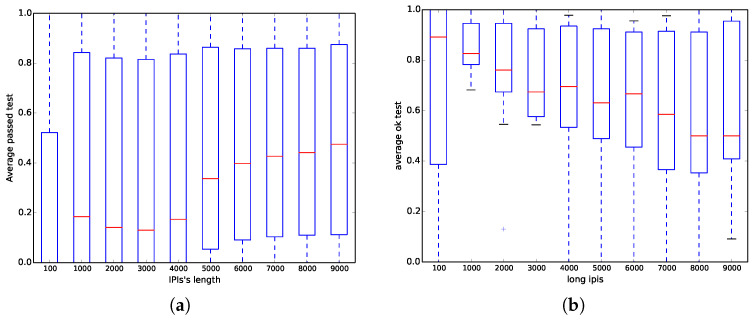
Distribution of the fraction of tests passed for the mitdb dataset as a function of the number of bits used. (**a**) ENT suite; and (**b**) NIST STS suite.

On the one hand, we have run all ENT tests (six out of six) for all databases with different samples per signal of each one of the subjects; however, here, we have only focused on the mitdb database because it has been commonly used in the literature. Figure 4a shows that when the length of the IPIs (number of bits in the file) is less than 6000 bits, the probability of success is less than 0.5 on average, whereas when the length is greater than 6000, the probability is between 0.1 and 0.8. Those results corroborate the same results previously obtained in Table 4, where the chi square test achieves 0% success, whereas the optimum compression test nearly has 100% success.

On the other hand, we have run all NIST STS tests (15 out of 15) for all databases with different IPI with respect to the median. Nevertheless, similar to the ENT experiment, we have only focused on the mitdb test instead of the rest of the databases. Contrary to the results obtained in Option a) in [6], Figure 4b shows that when the length of the IPI (number of bits in the file) increases, the results are worse, and even when the length is higher than 7000 bits, the probability of being successful is close to 0.5.

After analyzing Table 7 carefully, where the average of the results can be seen, we extract the following information:

- When the median number of IPIs is higher than 1800, then the databases achieve extremely poor results (two passed tests out of 15 in the worst case) in the NIST STS. Examples of these databases are vfdb, szdb, slpdb, mghdb, edb, apnea-ecg and shareedb.
- When the median number of IPIs is between 1800 and 415, then the databases are on the borderline of passing (at least) half of the NIST STS. Examples of these databases are svdb, cudb, stdb, qtdb, mitdb and nstdb. There is also one exception to this rule: aami-ec13, which has a median of 48.5 IPIs, and it achieves a 33.3% (five passed tests out of 15), which is similar to the results of svdb.
- When the median number of IPIs is between 415 and 37, the databases achieve extremely good results (14 passed tests out of 15 in the best case) in the NIST STS. Examples of these databases are cdb, twadb, pbdb, iafdb, cebsdb. As before, there is an exception to this rule: aami-ec13, which has a median of 48.5 IPIs, and it only passes five out of 15 tests.

We have tested 19 public databases from the Physionet repository. This has recently become a common practice in security proposals, and mitdb has been used as a starting point for authentication- and security-based protocols. According to the results presented in this work, we can claim that mitdb is not the best database for this purpose, but cebsdb is. However, other tests such as Diehard were impossible to be run with these databases because of the length of the signals; Diehard needs binary files that usually go from 10 to 12 million bytes.

## 4. Conclusions

In this work, we have addressed the random number generation issue by using heart signals; in particular, ECG records are used. Some authors have claimed that the four LSBs of the IPI values have a certain entropy level. Despite this, we have proven they have some entropy degree, and we have also showed that the ECG records and, consequently, the IPI values derived from them should not be considered a good source of randomness only by observing that value. We have used both the ENT and NIST STS test suites to evaluate the randomness property of 19 public and well-known ECG databases, and the results point to the fact that IPIs values are not as random as supposed. The database that achieves better results is cebsdb (healthy volunteers records) instead of mitdb (arrhythmia record), which is the most common database used in the literature. The use of the cebsdb database seems more appropriate since users do not suffer any medical condition and no defect (or bias) is a priori expected in the signals; in addition, the size of the database is more appropriate.

The results obtained through the in-depth analysis conducted clearly point to two conclusions: (1) a short burst of bits derived from an ECG record may seem random; but, (2) large files derived from long ECG records should not be used for security purposes (e.g., key generation algorithms). These conclusions should be taken with caution since these are conditioned on: (1) the IPI extraction algorithm described in Section 3.2; and, (2) the 19 public databases studied. Finally, we highlight here that all the necessary scripts to reproduce our experiments are publicly available (https://github.com/aylara/Random_ECG).

As future work, we plan to extend this analysis to other biological signals like PPG or EEG.

## Figures and Tables

**Table 1:** Datasets and number of test runs used by related work.

Work	Dataset	Randomness Test
[25]	50 subjects from the MIMICII Waveform	Shannon’s Entropy
[51]	99 subjects from a private dataset	NIST STS (5/15)
[52]	50 subjects from a private dataset	NIST STS (15/15)
[53]	Not specified	NIST STS (6/15)
[6]	47 subjects from mitdb; 290 from ptdb; 250 from mghdb	NIST STS (8/15)
[28]	mitdb (no info is given)	Shannon’s entropy
[30]	mitdb (no info is given)	Shannon’s entropy
[8]	mitdb (no info is given)	ENT
[29]	mitdb (no info is given)	Rényi’s entropy
[47]	PhysioNet ${}^{1}$	NIST STS (9/15)
[26]	84 subjects from a private dataset and European ST-T	NIST STS (5/15)
[27]	18 subjects from MIT-BIHand 79 from the European ST-T	NIST STS (10/15)

${}^{1}$ It is not specified in the paper.

**Table 2:** The 19 datasets used in this work. For each dataset, the table provides the number of records (subjects), the sampling frequency, the median value of IPIs per database and the pathology (if any) of the subjects involved in each dataset.

Dataset	#Records	Frequency (Hz)	Median (IPIs)	Pathology
cebsdb [31,55,56]	54	5000	175	Healthy volunteers
ptbdb [57]	545	1000	68	Myocardial problems and healthy controls
twadb [58]	5	500	87	Myocardial problems
iafdb [59]	5	1000	37	Atrial fibrillation or flutter
cdb [60]	53	250	12	Holter recordings
nstdb [61]	14	360	1246	Physically-active volunteers
mitdb [62]	46	360	1113	Arrhythmia
qtdb [63]	104	250	520.5	Holter recordings
stdb [64]	28	360	1243	Stress tests
cudb [65]	9	250	415	Ventricular problems
aami-ec13 [66]	10	720	48.5	Tachycardia
svdb [67]	47	128	1192	Partial epilepsy
vfdb [68]	17	250	1800	Tachycardia
szdb [69]	7	200	4439	Partial epilepsy
slpdb [70]	17	250	11,517	Sleep apnea syndrome
edb [59]	90	250	4405	Myocardial and hypertension
mghdb [71]	202	360	2426	Unstable patients in critical care units
apnea-ecg [72]	77	100	15,786	Tachycardia
shareedb [73]	23	128	46,910	Hypertension

**Table 3:** ENT tests: optimal values, thresholds used to consider that a sequence passes the test and results obtained for a counting sequence.

Test	Optimal Value	Threshold	Counter
Entropy	1.0	>0.85	0.99
Optimum compression	<0%	<5%	0%
Chi square	$5\%<{\tilde{\chi }}^{2}<$ 95%	$5\%<{\tilde{\chi }}^{2}<$ 95%	1%
Arithmetic mean	0.5	0.4 $<\bar{x}<$ 0.6	0.46
Monte Carlo value for π	error = 0%	error < 5%	12.38%
Serial correlation coefficient	0	$<10^{-1}$ or $<10^{-2}$	0.012

**Table 4:** Results of the ENT tests expressed as the percentage of subjects that pass each test per database.

Dataset	Entropy	Optimum Compression	Chi Square	Arithmetic Mean	Monte Carlo Value for π	Serial Correlation
cebsdb	100%	100%	0%	50%	10%	60%
ptbdb	99.82%	100%	0%	97.98%	22.20%	99.63%
twadb	100%	100%	0%	80%	0%	100%
iafdb	100%	100%	0%	100%	40%	100%
cdb	100%	100%	0%	81.13%	1.89%	96.23%
nstdb	100%	100%	0%	92.86%	35.71%	100%
mitdb	100%	100%	0%	97.83%	47.83%	97.83%
qtdb	99.04%	100%	0%	96.15%	38.46%	100%
stdb	100%	100%	0%	100%	35.71%	100%
cudb	100%	100%	0%	44.44%	11.11%	100%
aami-ec13	80%	100%	0%	50%	10%	60%
svdb	100%	100%	0%	97.87%	42.55%	97.87%
vfdb	83%	100%	0%	17%	6%	94%
szdb	85.71%	100%	0%	85.71%	71.43%	85.71%
slpdb	100%	100%	0%	100%	74.47%	100%
edb	98.89%	100%	0%	98.89%	60%	100%
mghdb	72.28%	100%	0%	59.41%	22.28%	86.14%
apnea-ecg	75.32%	100%	0%	62.34%	29.87%	81.82%
shareedb	95.65%	100%	0%	95.65%	55.52%	100%

**Table 5:** NIST STS requirements in terms of length [76].

Test Name	*n*	*m* or *M*
Frequency (Monobit)	n≥100	-
Frequency Test within a Block	-	20≤M≤n/100
Run	n≥100	-
Longest Run of Ones in a Block		
Binary Matrix Rank	n≥38,912	-
Discrete Fourier Transform (Spectral)	n≥1000	-
Non-Overlapping Template Matching		2≤m≤21
Overlapping Template Matching		1≤m≤n
Maurer’s “Universal Statistical” Test		1≤m≤n
Linear Complexity	$n\geq 10^{6}$	500≤M≤5000
Serial		$3\leq m\leq \lfloor log_{2}n\rfloor -3$
Approximate Entropy		$m\leq \lfloor log_{2}n\rfloor -6$
Cumulative Sums	n≥100	
Random Excursions	$n\geq 10^{6}$	
Random Excursions Variant	$n\geq 10^{6}$	

**Table 6:** Results of the NIST STS tests expressed as the percentage of subjects that pass each test.

Dataset	Monobit Frequency	Block Frequency	Runs	Longest Run Ones	Binary Matrix Rank	Spectral	Non Overlapping Template Matching	Overlapping Template Matching	Universal Statistic	Linear Complexity	Serial	Approximate Entropy	Cumulative Sums	Random Excursions	Random Excursions Variant
cebsdb	94%	81%	85%	87%	98%	100%	96%	72%	100%	20%	85%	96%	96%	98%	100%
ptbdb	89%	89%	89%	89%	85%	92%	98%	84%	1%	0%	100%	85%	98%	99%	65%
twadb	80%	60%	80%	100%	80%	80%	100%	80%	20%	0%	100%	60%	100%	80%	80%
iafdb	100%	100%	100%	80%	100%	100%	100%	100%	20%	0%	100%	100%	80%	100%	40%
cdb	94%	25%	92%	94%	100%	94%	96%	81%	0%	0%	100%	77%	100%	100%	0%
nstdb	14%	21%	7%	64%	21%	50%	71%	57%	86%	57%	7%	93%	93%	71%	100%
mitdb	46%	33%	33%	50%	35%	59%	91%	52%	89%	39%	9%	87%	96%	85%	100%
qtdb	47%	41%	44%	54%	25%	53%	92%	56%	89%	0%	0%	77%	98%	81%	95%
stdb	50%	7%	29%	54%	21%	21%	64%	32%	64%	21%	4%	71%	100%	32%	100%
cudb	0%	11%	0%	56%	11%	22%	11%	67%	67%	0%	11%	56%	100%	22%	78%
aami-ec13	10%	20%	30%	40%	20%	10%	90%	90%	0%	0%	100%	60%	100%	0%	40%
svdb	23%	11%	19%	28%	6%	9%	77%	43%	77%	21%	0%	77%	100%	28%	94%
vfdb	29%	12%	12%	41%	18%	12%	29%	29%	88%	18%	0%	71%	100%	24%	100%
szdb	14%	0%	0%	29%	0%	0%	43%	29%	71%	0%	0%	86%	100%	0%	86%
slpdb	24%	0%	6%	35%	6%	12%	76%	12%	24%	0%	0%	76%	94%	6%	94%
edb	23%	1%	14%	29%	3%	7%	62%	21%	43%	9%	0%	86%	100%	2%	94%
mghdb	22%	14%	14%	33%	11%	12%	35%	34%	34%	7%	19%	57%	99%	15%	60%
apnea-ecg	5%	4%	4%	17%	1%	0%	27%	26%	23%	0%	9%	68%	96%	1%	75%
shareedb	9%	0%	0%	4%	0%	0%	17%	0%	0%	0%	0%	48%	100%	0%	96%
**Average**	36.8%	21.0%	26.3%	52.6%	26.3%	42.1%	68.4%	52.6%	47.3%	5.2%	31.5%	94.7%	100%	42.1%	84.2%

**Table 7:** Characteristics vs. success rate datasets.

Dataset	ENT	NIST STS	Avg. No. Samples	Median (IPI)	Pathology
cebsdb	66.6%	93.3%	4,968,780	175	Healthy volunteers
ptbdb	66.6%	86.6%	108,818	68	Myocardial problems and Healthy controls
twadb	66.6%	86.6%	59,770	87	Myocardial problems
iafdb	66.6%	80.0%	19,707,034	37	Atrial fibrillation or flutter
cdb	66.6%	73.3%	5,120	12	Holter recordings
nstdb	66.6%	66.6%	650,000	1246	Physically active volunteers
mitdb	66.6%	60.0%	650,000	1113	Arrhythmia
qtdb	66.6%	60.0%	224,999	520.5	Holter recordings
stdb	66.6%	46.6%	624,166	1243	Stress tests
cudb	50.0%	40.0%	127,232	415	Ventricular problems
aami-ec13	66.6%	33.3%	55,522	48.5	Tachycardia
svdb	66.6%	33.3%	230,400	1192	Partial epilepsy
vfdb	50.0%	26.6%	525,000	1800	Tachycardia
szdb	83.3%	26.6%	17,245,701	4439	Partial epilepsy
slpdb	83.3%	26.6%	4,188,530	11,517	Sleep apnea syndrome
edb	83.3%	26.6%	1,800,000	4405	Myocardial and hypertension
mghdb	66.6%	20.0%	1,479,358	2426	Unstable patients in critical care units
apnea-ecg	66.6%	20.0%	11,930	15,786	Tachycardia
shareedb	83.3%	13.3%	10,553,116	46,910	Hypertension

## Appendix A. Random Tests

### Appendix A.1. ENT

ENT [54] is a battery of tests used to evaluate pseudorandom number generators. The program reports the overall randomness results after running five different statistical tests: entropy, chi square, arithmetic mean, Monte Carlo and serial correlation coefficient. A brief description of each test is given next.

**Entropy test:** This test measures the amount of information of the sequence, expressed as a number of bits per character. The higher the result, the more random the sequence is considered.

**Chi square test:** The chi square test is one of the most commonly-used tests and is extremely sensitive to errors in pseudorandom sequence generators. The test computes the chi-square distribution for the input stream of bits and provides the result both as an absolute number and a percentage indicating how frequently a truly random sequence would exceed the calculated value.

**Arithmetic mean:** The value of this test indicates the result of adding up all bytes in the sequence and dividing this by the sequence length (in bytes). The closer the result is to 128, the more random the results.

**Monte Carlo value for π:** This test estimates the value of π through a standard Monte Carlo method using the input sequence, which is considered random when the computed value is close to the true value of π. The test outputs both the estimated value of π and the error.

**Serial correlation coefficient:** This test attempts to capture correlations in the sequence by checking how much each byte in the stream depends on the previous one. The closer the result is to zero, the more random the sequence.

### Appendix A.2. NIST STS

The NIST STS test suite [12] is a set of fifteen statistical tests to evaluate random and pseudo-random number generators used in cryptographic applications. These tests are often used as a first step in spotting low-quality generators, but they are by no means a substitute for cryptanalysis. In other words, successfully passing all tests does not guarantee that the generator is strong enough.

All tests take as input a sequence of (binary) numbers and return a *p*-value that is then used to assess whether the sequence passed or not each test. In the following, we briefly describe each test in turn.

**Frequency (monobit) test:** This is one of the simplest tests, which checks if the input sequence has a balanced number of ones and zeroes (i.e., if the distribution of bits is uniform).

**Frequency test within a block:** This test is an extension of the frequency monobit test, which can be considered as a particular case with the block size *M* equal to one. For values M>1, this test checks if the frequency of ones in an *M*-bit block is approximately M/2.

**The runs test:** This test measures whether the number of runs of ones and zeroes of various lengths is as would be expected for a truly random sequence [12]. A run is a consecutive sequence of bits with the same value. The test returns a *p*-value per block length.

**Longest-run-of-ones in a block:** This test checks the length of the longest run of ones in a previously-defined block with length *M* and compares it with the expected value for a truly random sequence.

**The binary matrix rank test:** This test generates m×n binary matrices over GF(2) using the values of the input sequence (each row of a matrix is a substring of the sequence) and checks whether the ranks are linearly dependent.

**Discrete Fourier transform (spectral) test:** This test calculates the discrete Fourier transform of each subsequence of bits and computes the peaks, which might reveal patterns in the original sequence. The test uses a threshold $t=\sqrt{log(1/0.05)n}$, where *n* is the length of the sequence. If the number of peaks is at most 5%, the sequence can be considered as random.

**Non-overlapping template matching test:** In this test, the random sequence is split into *M* substrings of length *l*. The test seeks the number of occurrences of a given template. If a pattern is found, the algorithm resets the substring *M* to the bit after the found pattern, otherwise *M* is reset to the next bit.

**The overlapping template matching test:** This test is identical to the non-overlapping template matching test, but using overlapping substrings (i.e., using a sliding window that advances 1 bit at a time).

**Maurer’s “universal statistical” test:** The purpose of this test is to detect if the sequence can be significantly compressed without loss of information. One of the main drawbacks of this test is that it requires a substantially long sequence for the result to be relevant.

**The linear complexity test:** This test computes the linear complexity of the input sequence. If the value is too short, the sequence is not considered random enough.

**The serial test:** The focus of this test is to calculate the frequency of all possible overlapping *M*-bit patterns in the whole sequence. That is, each *M*-block should have the same probability of appearing as any other *M*-bit pattern.

**The approximate entropy test:** This test is focused on the frequency of all possible overlapping *m*-bit patterns in a sequence. In short, this test compares the frequency of two adjacent lengths (*m* and m+1) to the expected result for a random sequence.

**The cumulative sums test:** In this test, zeroes are converted to negative ones, and ones remain the same. This test is based on the maximum distance from zero of a random walk defined by the cumulative sum of the sequence. For a random sequence, the cumulative sum should be close to zero.

**The random excursions test:** This test calculates the number of cycles having exactly *K* visits in a cumulative sum random walk, which is derived from partial sums.

**The random excursions variant test:** This test is similar to the random excursion test, but in this case, the goal is to detect deviations from the expected number of visits to various states in the random walk.
